# A Probabilistic and Highly Efficient Topology Control Algorithm for Underwater Cooperating AUV Networks

**DOI:** 10.3390/s17051022

**Published:** 2017-05-04

**Authors:** Ning Li, Baran Cürüklü, Joaquim Bastos, Victor Sucasas, Jose Antonio Sanchez Fernandez, Jonathan Rodriguez

**Affiliations:** 1 Escuela Técnica Superior de Ingeniería y Sistemas de Telecomunicación, Campus Sur Universidad Politécnica de Madrid (UPM), 28031 Madrid, Spain; j.sanchez@upm.es; 2 Division of Intelligent Future Technologies, School of Innovation, Design and Engineering, Malardalen University, 721 23 Västerås, Sweden; baran.curuklu@mdh.se; 3 Instituto de Telecomunicações, Campus de Santiago, 3810-193 Aveiro, Portugal; jbastos@av.it.pt; 4 Universidade de Aveiro, Campus Universitario de Santiago, 3810-193 Aveiro, Portugal; vsucasas@ua.pt (V.S.); jonathan@ua.pt (J.R.)

**Keywords:** topology control, underwater network, AUV, probabilistic, transmission power adjustment

## Abstract

The aim of the Smart and Networking Underwater Robots in Cooperation Meshes (SWARMs) project is to make autonomous underwater vehicles (AUVs), remote operated vehicles (ROVs) and unmanned surface vehicles (USVs) more accessible and useful. To achieve cooperation and communication between different AUVs, these must be able to exchange messages, so an efficient and reliable communication network is necessary for SWARMs. In order to provide an efficient and reliable communication network for mission execution, one of the important and necessary issues is the topology control of the network of AUVs that are cooperating underwater. However, due to the specific properties of an underwater AUV cooperation network, such as the high mobility of AUVs, large transmission delays, low bandwidth, etc., the traditional topology control algorithms primarily designed for terrestrial wireless sensor networks cannot be used directly in the underwater environment. Moreover, these algorithms, in which the nodes adjust their transmission power once the current transmission power does not equal an optimal one, are costly in an underwater cooperating AUV network. Considering these facts, in this paper, we propose a Probabilistic Topology Control (PTC) algorithm for an underwater cooperating AUV network. In PTC, when the transmission power of an AUV is not equal to the optimal transmission power, then whether the transmission power needs to be adjusted or not will be determined based on the AUV’s parameters. Each AUV determines their own transmission power adjustment probability based on the parameter deviations. The larger the deviation, the higher the transmission power adjustment probability is, and vice versa. For evaluating the performance of PTC, we combine the PTC algorithm with the Fuzzy logic Topology Control (FTC) algorithm and compare the performance of these two algorithms. The simulation results have demonstrated that the PTC is efficient at reducing the transmission power adjustment ratio while improving the network performance.

## 1. Introduction

In the near future, the ocean will supply a substantial part of human and industrial needs: the oil and gas industry will move into deeper waters, the renewable energy will be harvested from sea, as well as many other innovative practices will become common. Furthermore, minerals such as cobalt, nickel, copper, rare earths, silver, and gold will be mined from the seafloor. One major challenge in this context is how to develop systems and solutions which can guarantee a sustainable development of the maritime activities so that this fragile habitat is protected for future generations. To this end, new offshore and port infrastructures need to be built, maintained, and repaired when necessary. Ocean monitoring and underwater exploration are not easy tasks, because the ocean is large and most of the underwater environment is still unknown to us. In addition, due to the high pressure in deep water, it is not suitable for people to work for long time under such conditions, which in several cases makes any human intervention impracticable. Thus, there is an obvious requirement for solutions that can assist humans in underwater mission. For this reasons, the Smart and Networking Underwater Robots in Cooperation Meshes (SWARMs) project (which can be found at http://swarms.eu/) has been proposed to provide feasible solutions for these kinds of issues. The aim of the SWARMs project is to make autonomous underwater vehicles (AUVs), remote operated vehicles (ROVs) and unmanned surface vehicles (USVs) further accessible and useful, which includes: (1) enabling AUVs/ROVs to work in a cooperative mesh thus opening up new applications and ensuring reusability; (2) increasing the autonomy of AUVs/USVs and improving the usability of ROVs for the execution of simple and complex tasks. Therefore, for achieving the cooperation and communication between different AUVs, the AUVs must be able to exchange messages between each other, which signifies an efficient and reliable communication network is necessary for SWARMs. 

Since radio frequency (RF) waves are seriously attenuated in the underwater environment [1], underwater AUVs use acoustic waves rather than RF waves to communicate with each other [2]. In SWARMs, for providing an efficient and reliable communication network to facilitate mission execution, one of the important and necessary issues is the topology control of the underwater cooperating AUVs network. The reasons are: (1) to achieving their missions, underwater AUVs keep moving along the pre-defined paths, in which case, the topology of an underwater AUV cooperation network changes frequently; (2) since the transmission speed of acoustic waves is much smaller than that of RF waves, the transmission delay in an Underwater Cooperating AUVs Network (UCAN) is more critical than that in traditional RF-based wireless sensor networks (WSNs) [3,4]; (3) due to the fact the bandwidth of the underwater acoustic channel is very limited, the congestion in such a kind of channel could be severe [5], which means the control messages of an underwater cooperating AUV network should be reduced as much as possible; (4) the multipath interference and the Doppler spreading are serious in the underwater environment. Although many topology control algorithms have been proposed for conventional WSNs, considering the control cost, these topology control algorithms are not efficient in an underwater cooperating AUVs network where the AUVs move frequently. For instance, in traditional topology control algorithms, once the current transmission power is not equal to the optimal one, which is calculated based on optimal algorithms (such as those described in [6,7,8,9]), then the nodes need to adjust their transmission power. In a network where the network topology changes slightly or the network resources are abundant, the above mentioned approach is effective. However, for the UCAN, in which the network resources are limited and the network topology changes frequently, this approach is not as efficient as in a static network. The heavy control cost caused by AUVs’ mobility will deteriorate the network performance greatly. Therefore, it is necessary to investigate how to reduce the control cost in order to improve the network performance of underwater cooperating AUV networks. 

Additionally, for an underwater cooperating AUV network, once the current transmission power of an AUV is not equal to the optimal transmission power which is calculated based on optimal algorithms (such as interference-based, transmission delay-based, energy consumption- based, etc.), considering the other parameters of the AUV, adjusting the transmission power may not be a good strategy to improve the network performance. This means it is necessary to accept a tradeoff between improving the network performance in one aspect and keeping the functionality of network (such as, network connectivity). For instance, when the optimal transmission power of AUV is $P^{*}$ and the current transmission power is P, if $P\approx P^{*}$ (which means the difference between *P* and $P^{*}$ is small enough), then even if the current transmission power does not equal the optimal one, optimization rules do not need to be applied when energy consumption and network congestion are taken into account. This tradeoff is useful for an underwater cooperating AUV network. Moreover, in an underwater cooperating AUV network, whether the transmission power needs to be adjusted or not also relates to other network parameters. For example, if $P\ll P^{*}$, but the residual energy of an AUV is small, in this case, it is better to not increase the transmission power in order to prolong the lifetime of this AUV. 

Motivated by these facts, in this paper, we propose a new topology control algorithm called the probabilistic topology control (PTC) algorithm for underwater cooperating AUV networks, which is based on the value of an AUV’s residual energy, queue length, current transmission power, and number of neighbors to determine the transmission power adjustment probability of the AUV. In PTC, when the transmission power is not equal to the optimal one, the AUVs do not adjust their transmission power immediately. The deviations of each AUV’s residual energy, current transmission power, queue length, and number of neighbors are used to calculate each parameter’s adjustment probability through a fuzzy logic algorithm. The larger the deviation, the larger the adjustment probability is. The probabilities are $\rho_{P}$, $\rho_{Q}$, $\rho_{E}$, and $\rho_{n}$, respectively. The maximum adjustment probability will be chosen as the transmission power adjustment probability of an AUV. Based on these innovations, the PTC algorithm can improve the network performance greatly; In particular it can reduce the transmission power adjustment ratio of the network. Note that the PTC algorithm can combine with any power control algorithm to reduce the transmission power adjustment ratio. The main contributions of this paper are as follows:

- We propose the definition of transmission power adjustment probability. Based on this definition, we propose the probabilistic topology control (PTC) algorithm for underwater cooperating AUV networks. In PTC, when the current transmission power does not equal the optimal one, whether an AUVs needs to adjust its transmission power or not will be decided based on the parameters of this AUV;
- We propose the definition of transmission power adjustment ratio for topology control algorithm. Based on this definition, we analyze the properties of PTC algorithm on reducing the transmission power ratio of the network;
- Combining with the fuzzy-logic topology control (FTC) algorithm, in this paper, we compare the performance of the PTC-based FTC algorithm and the standalone FTC algorithm. The simulation results demonstrate the effectiveness of the PTC algorithm in improving the network performance.

The rest of this paper is organized as follows: in Section 2, we introduce the related works published in recent years; in Section 3, we first introduce the channel model, the path loss model, and the network model; then we propose the calculation of parameter deviations and the transmission power probability of AUVs; finally, based on the conclusions introduced above, we propose the PTC-based FTC algorithm; Section 4 presents the simulation results of the performance of PTC-FTC algorithm and FTC algorithm; Section 5 concludes the work in this paper. 

## 2. Related Works

There are many topology control algorithms have been proposed in recent years for both the underwater and terrestrial WSNs. In the following subsections, we will review these algorithms briefly.

### 2.1. The Topology Control Algorithms of RF-Based WSNs

For improving the connectivity and the reliability of WSNs, in [6], the authors proposed a novel fuzzy-logic topology control (FTC) algorithm to achieve any desired average node degree by adaptively changing the transmission power. The FTC algorithm does not rely on location information of neighbors and is constructed from the training data set to facilitate the design process. In [7], for reducing energy consumption and end to end delay of WSNs, the authors proposed an optimization problem for energy consumption in WSNs, in which the topology control and the network-coding based multi-cast are combined together. This optimization problem is transformed into a convex problem which offers numerous theoretical and conceptual advantages. In this algorithm, the Karush-Kuhn-Tucker optimality conditions are presented to derive analytical expressions of the globally optimal solution. By these innovations, the performance of energy consumption and end to end delay was improved. In [8], the authors investigated a dynamic topology control scheme to improve the network lifetime for WSNs in the presence of selfish sensors, and propose a non-cooperative game-aided topology control approach to design energy-efficient and energy balanced network topologies dynamically. The nodes in the topology control game try to minimize their unwillingness to construct a connected network according to their residual energy and transmission power. In [9], considering the lossy links which can only provide probabilistic connectivity in network, the authors propose the probabilistic topology control (PTC). In PTC, the network connectivity is metered by network reachability and is defined as the minimal upper limit of the end-to-end delivery ratio between any pair of nodes in network. The PTC algorithm can find a minimal transmission power for each node while the network reachability is above a given application-specified threshold. The adaptive disjoint path vector (ADPV) algorithm has been proposed for heterogeneous WSNs in [10]. In ADPV, the algorithm is divided into two phases: single initialization phase and restoration phase. The restoration phase utilizes the alternative routes that are computed in the initialization phase with the help of a novel optimization algorithm which is based on the well-known set-packing problem. The simulation results demonstrate that the ADPV is superior in preserving super node connectivity. The authors in [11] consider that topology control has never achieved breakthroughs in real world deployment; moreover, the authors identify five practical obstacles of topology control algorithms at present. To address these obstacles, the authors propose a re-usable framework for implementation and evaluation of topology control. In [12] the authors propose the concept of a disjoint path vector (DPV) algorithm for a heterogeneous network in which the large number of sensor nodes have limited energy and computing capability and there are several supernodes with limited energy and unlimited computing capability. The DPV algorithm addresses the *k*-degree any-cast topology control problem where the main objective is to assign each sensor’s transmission range such that each node has at least *k*-vertex-disjoint paths to super nodes and the total power consumption is minimized. The resulting topologies are tolerant up to *k-1* node failures in the worst case. In [13], to enhance the energy efficiency and reduce the radio interference in WSNs, the authors propose a new distributed topology control algorithm. In this algorithm, each node makes local decisions about its transmission power and the culmination of these local decisions produces a network topology that preserves global connectivity. The main idea of this topology control algorithm is the novel Smart Boundary Yao Gabriel Graph (SBYaoGG) and the appropriate optimizations to ensure that all links in network are symmetric and energy efficient. The more recent researches on topology control can be found in [14,15,16,17,18]. Moreover, detailed introductions and comparisons between different topology control algorithms can be found in reviews, such as [19,20,21]. 

### 2.2. The Topology Control Algorithms of Underwater WSNs

The topology control algorithms of underwater WSNs are not as extensively investigated as those of terrestrial WSNs. In [22], considering the signal irregularity phenomenon can affect network performance, especially in underwater environments, the authors constructed an authentic signal irregularity model which can easily be degenerated into a variety of special cases. Based on this model, three representative topology control objectives are concluded in this work. In [23], two topology control algorithms are proposed for underwater WSNs: improved Distributed Topology Control (iDTC) and Power Adjustment Distributed Topology Control (PADTC). These two algorithms can increase network throughput while conserving energy at the same time. The algorithms guarantee the delivery of data by dealing with the communication void problem in geographic opportunistic routing. In [24], the authors investigate scale-free underwater WSNs. The algorithm begins with a scale-free network model for calculating the edge probability, which is used to generate an initial topology randomly. Subsequently, a topology control strategy based on complex network theory is put forward to construct a double clustering structure, where there are two kinds of cluster-heads to ensure connectivity and coverage. Considering that using the Global Positioning System (GPS) may not be feasible in adverse underwater environments and the anchored sensor nodes towed by wires are prone to offset around their static positions which causes each node to move within a spherical crown surface, in [25], the authors proposes a mobility model for underwater WSNs and three representative topology control objectives are attained. Based on these objectives, the authors design a distributed radius determination algorithm for the mobility-based topology control problem. Due to the fact the coverage requirements in different regions are probably different in underwater environments, in [26], the authors proposed two algorithms for different coverage problems in underwater WSNs: a Traversal Algorithm for Different Coverage (TADC) and a Radius Increment Algorithm for Different Coverage (RIADC). The TADC adjusts the sensing radii at each round and the RIADC sets the sensing radii of nodes incrementally at each round. In [27], the authors illuminated network topology modeling from a routing viewpoint. The probabilistic multipath routing behavior which is driven by opportunistic routing protocols in underwater WSNs are modeled in this paper. Based on these models, the authors proposed the PCen centrality metric to measure the importance of underwater sensor nodes to the data transmission through opportunistic routing, which is aimed at identifying critical nodes that can be used to guide topology control solutions. 

## 3. Probabilistic Topology Control Algorithm

In this section, we will introduce the probabilistic topology control algorithm in detail. Note that although this algorithm shares its name with the one discussed in [9], these two topology control algorithms are totally different. 

### 3.1. Communication Network Architecture of SWARMs Project

**Figure 1:**
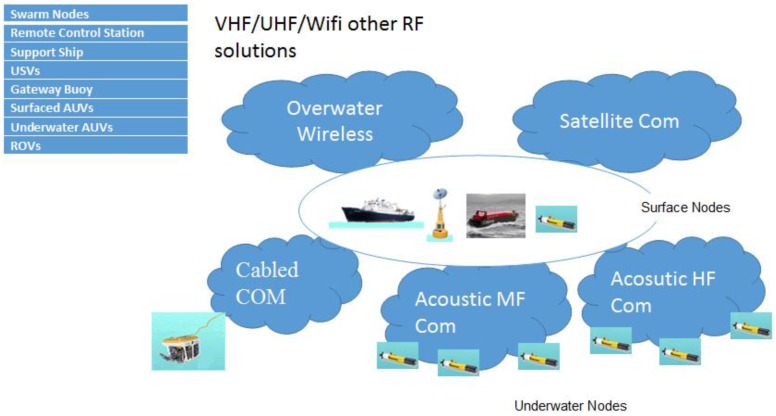
The architecture of the communication network in SWARMs project.

The architecture of the communication network used in the SWARMs project can be seen in Figure 1. In SWARMs project, the communication network has been divided into five different categories: (1) overwater RF wireless communication network; (2) satellite communication network; (3) cabled communication network; (4) acoustic MF communication network; (5) acoustic HF communication network. In this paper, we mainly focus on the acoustic MF communication network. Based on the architecture of the communication network, many use cases have been proposed in the SWARMs project; for instance, corrosion prevention in offshore installation, monitoring of chemical pollution, detection, inspection and traction of plumes, berm building, and seabed mapping, which can all be found at http://swarms.eu/usecases.html.

**Figure 2:**
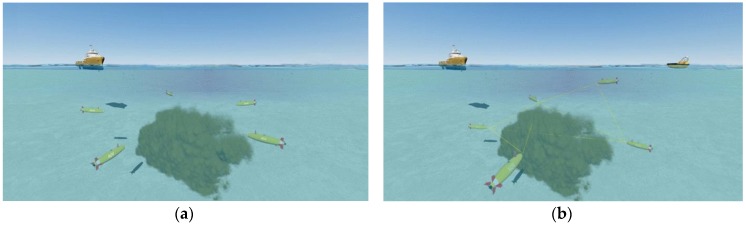
Use case of detection, inspection and traction of plumes in the SWARMs project: (**a**) AUVs tracking and detecting the plume; (**b**) AUVs sharing the information between each other.

In this paper, the topology control algorithm is designed for the detection, inspection and traction of plumes (see Figure 2). In this use case AUVs display two different kinds of movement pattern: (1) all AUVs in network move in a group with the same movement pattern as plumes; (2) in the interior of network, the AUVs move freely in the area where the plumes exist; moreover, for guaranteeing this area can be covered by the AUVs’ transmission area, the movement of AUVs should be able to guarantee that the AUVs are approximately uniformly distributed in the area where the plumes exist. These two kinds of movement are different. Considering the first kind of movement, since the movement of plumes is random, the AUVs must be able to detect the movements of plumes and trace them; in most cases, the movement of plumes under the water can be regarded as a group mobility problem and many mobility models can be used to describe this kind of movement, such as the reference point group mobility model [28], the nomadic community mobility model [29], the reference velocity group mobility model [30], etc. Consequently the first kind of movement of AUVs is similar to the movement of plumes. However, concerning the second movement, which is the inner-network movement, the AUVs can move in the area where the plumes exists freely; moreover, for guaranteeing this area can be covered by AUVs’ transmission area, the AUVs should be uniformly distributed in this area.

#### 3.1.1. The Parameters of the Underwater Environment

Considering the fact that different hydrological parameters have different effects on the communication performance of an underwater cooperating AUVs network, we present the hydrological parameters of the test location in this section. These hydrological parameters, including the water temperature, water salinity, and sound speed, are the average values of the test location in past decades. 

**Figure 3:**
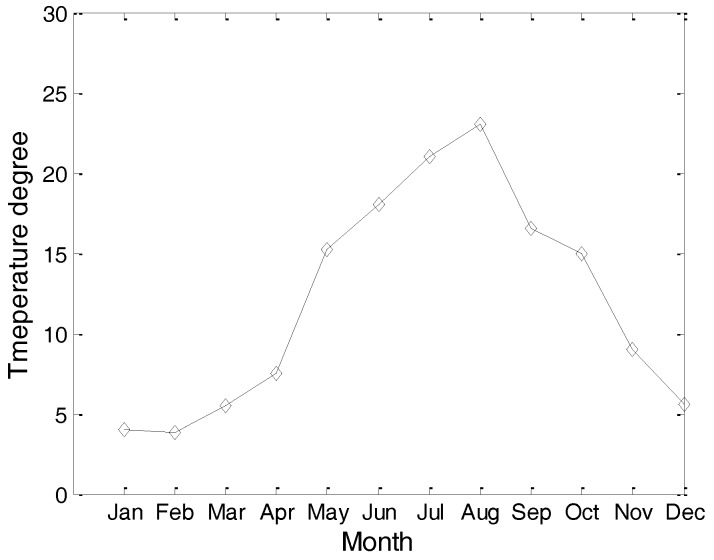
Average temperature in a year.

**Figure 4:**
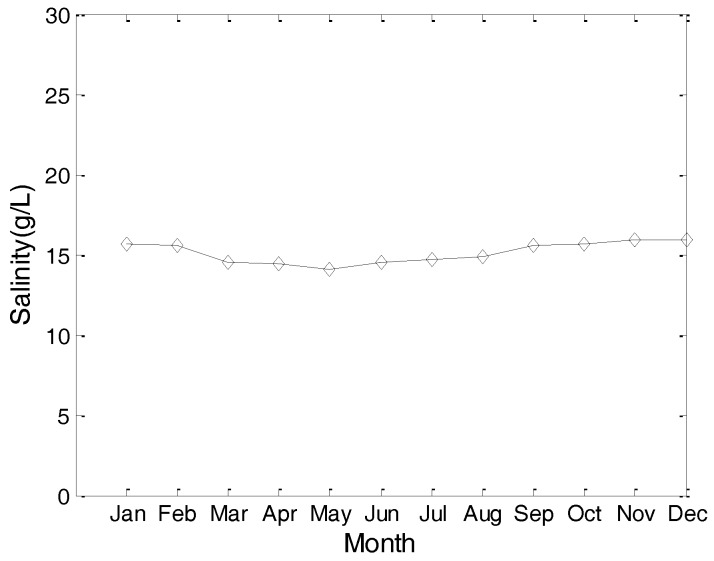
Average salinity in a year.

**Figure 5:**
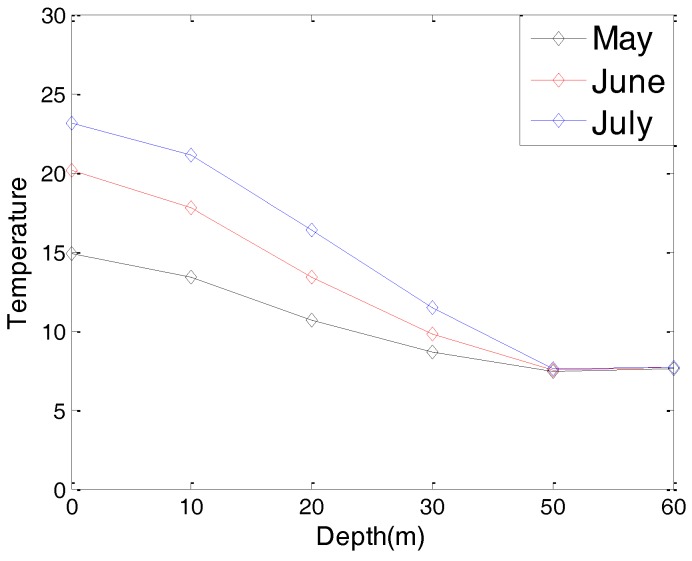
Average temperature with different water depths.

**Figure 6:**
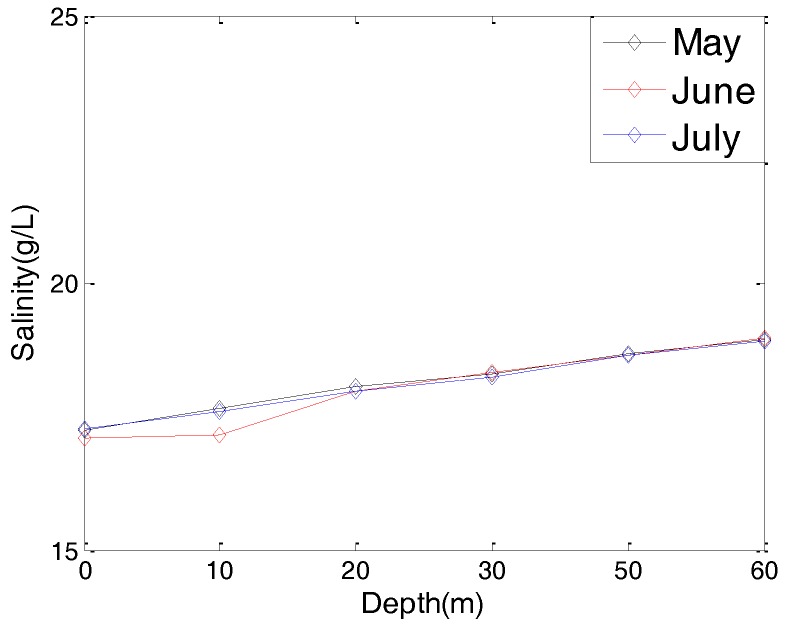
Average salinity with different water depths.

**Figure 7:**
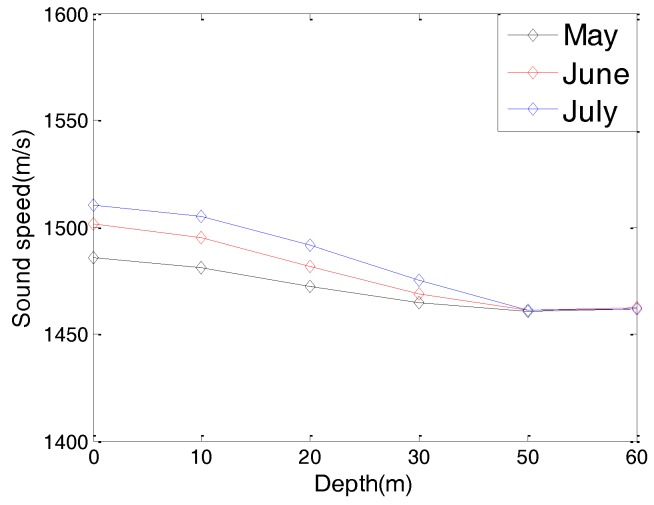
Average sound speed with different water depths.

Figure 3 and Figure 4 are the yearly average temperature and salinity, respectively, when the water depth is 10 m. Figure 5 and Figure 6 are the average temperature and salinity for different water depths and months. Figure 7 illustrates the average sound speed in different months with different water depths. 

#### 3.1.2. Hardware Parameters

In an underwater cooperating AUVs network, communication modules are equipped on the underwater AUVs to allow them to communicate with each other. Three different kinds of AUVs are used in SWARMs project, and the parameters of these AUVs can be found in Table 1.

The communication modules used in the SWARMs project include medium frequency (MF) modules and high frequency (HF) modules. The MF communication modules are used for data exchanges between different AUVs; the HF communication modules are used for point to point (P2P) communication between different AUVs and ROVs. In this paper, the topology control algorithm is mainly designed for MF communication networks. The MF communication modules used in the SWARMs project are the S2CR communication module. The module operates in the frequency band 18–34 kHz around a central frequency of 25 kHz with an efficient frequency bandwidth of 16 kHz. Sweep-spread carrier is used for data encoding in the S2CR module. The details of this communication module can be found in Table 2. These parameters will be used in our simulation.

#### 3.1.3. The Sweep-Spread Carrier Model

The S2CR module shown in Table 2 is built upon the sweep-spread carrier (S2C) technology [31]. In the following, we will introduce this technology under multipath environment and Doppler spreading environment briefly. 

##### Digital Signal with Sweep Spread Carrier

Assuming that the sweep spread carrier (S2-carrier) consists of a succession of sweeps with frequency variation from $\omega_{L}$ to $\omega_{H}$ within a time interval $T_{sw}$, and all the sweeps will be uniformly produced in a linear manner with rapid frequency variation following each other successively without any gap between them. Then the S2-carrier can be expressed as:

(1) $$c(t)=A_{c}e^{j(\omega_{L}(t-\left\lfloor \frac{t}{T_{sw}}\right\rfloor T_{sw})+m{\left(t-\left\lfloor \frac{t}{T_{sw}}\right\rfloor T_{sw}\right)}^{2})},$$

where $A_{c}$ is the amplitude; $m=\frac{\left(\omega_{H}-\omega_{L}\right)}{2T_{sw}}$ is a coefficient denoting the frequency variation rate; $\omega_{L}$ and $\omega_{H}$ denote the lowest and highest angular frequencies, respectively; $T_{sw}$ is the sweep duration; the term $\left\lfloor \frac{t}{T_{sw}}\right\rfloor$ denotes the operand for truncating the value to the nearest least integer, which is defined as:

(2) $$\left(t-\left\lfloor \frac{t}{T_{sw}}\right\rfloor T_{sw}\right)=\left\{\frac{t}{T_{sw}}\right\}T_{sw},$$

Equation (2) can be interpreted in Equation (1) as an actual cycle time with the cycle duration $T_{sw}$.

##### Signal with Sweep Spread Carrier under Multipath Channel

Based on the conclusions in Equations (1) and (2), the signal with S2C in a multipath channel can be calculated. Let the symbol *s*(*t*) be phase encoded data. The symbols are modulated onto the S2C, which is x(t)=s(t)⋅c(t). The signal is transmitted over a dispersive underwater channel. The part of the model which represents the water medium consists of a number of delay elements $\tau_{i}$ which denote the time intervals between two successive multipath arrivals, and a number of multiplication elements $V_{i}$ which takes possible attenuations on interfering multipath arrivals into account.

If both c(t) and s(t) have unit amplitudes, and every coefficient $V_{i}$ and delay element $\tau_{i}$ remain constant during the entire transmission time, then after propagation along different paths in an underwater medium, the signals received by a receiver can be calculated as:

(3) $$y(t)=V_{0}x(t)+\sum_{i}V_{i}x(t-\tau_{i})+n(t),$$

where x(t) is defined as above, and $x(t-\tau_{i})$ can be expressed as:

(4) $$x(t-\tau_{i})=s(t-\tau_{i})\cdot e^{j(\omega_{L}\left\{\frac{t-\tau_{i}}{T_{sw}}\right\}T_{sw}+m{\left(\left\{\frac{t-\tau_{i}}{T_{sw}}\right\}T_{sw}\right)}^{2})},$$

where n(t) is the white noise. It is evident that:

(5) $$\left\{\frac{t-\tau_{i}}{T_{sw}}\right\}T_{sw}=\left\{ \begin{array}{l} t_{c}-\tau_{ci},\;t_{c}\geq \tau_{ci}\\ T_{sw}+t_{c}-\tau_{ci},\;t_{c}<\tau_{ci} \end{array}\right.,$$

where $t_{c}=\left\{\frac{t}{T_{sw}}\right\}T_{sw}$ is the cycle time defined in Equation (2), and $\tau_{ci}=\left\{\frac{\tau_{i}}{T_{sw}}\right\}T_{sw}$ is a fractional part of time delay related to sweep duration $T_{sw}$. Thus, every delayed arrival represented in the second member of Equation (3) can be rewritten as:

(6) $$x(t-\tau_{i})=\left\{ \begin{array}{l} s(t-\tau_{i})\cdot e^{j(\omega_{L}(t_{c}-\tau_{ci})+m{\left(t_{c}-\tau_{ci}\right)}^{2})},\;t_{c}\geq \tau_{ci}\\ s(t-\tau_{i})\cdot e^{j(\omega_{L}(T_{sw}+t_{c}-\tau_{ci})+m{\left(T_{sw}+t_{c}-\tau_{ci}\right)}^{2})},\;t_{c}<\tau_{ci} \end{array}\right.,$$

After transformation of Equation (6), each delayed arrival can be written as:

(7) $$x(t-\tau_{i})=s(t-\tau_{i})\cdot e^{j(\omega_{L}t_{c}+mt_{c}^{2})}\cdot e^{j(-\Delta \omega_{i}t_{c}+\phi_{i})},$$

where $\Delta \omega_{i}=\left\{ \begin{array}{l} 2m\tau_{ci},\;t_{c}\geq \tau_{ci}\\ -2m(T_{sw}-\tau_{ci}),\;t_{c}<\tau_{ci} \end{array}\right.$ is the frequency deviation of the *i*-th multipath arrival caused by delay $\tau_{i}$, and $\phi_{i}=\left\{ \begin{array}{l} (m\tau_{ci}-\omega_{L})\tau_{ci},\;t_{c}\geq \tau_{ci}\\ (\omega_{L}+\omega_{L}-2m\tau_{ci})\frac{T_{sw}-\tau_{ci}}{2},\;t_{c}<\tau_{ci} \end{array}\right.$ is the phase of the *i*-th multipath arrival.

The term with *i* = 0 in Equation (3) represents an attenuated version of the original signal, and the other term is the multipath diversity of its delayed, attenuated and frequency shifted reproductions. The most important feature of Equation (7) is that at any instant all the interfering multipath arrivals have different frequencies spaced by $\Delta \omega_{i}$ from each other. 

##### Signal with Sweep Spread Carrier under Doppler Spreading

The same can be shown for time-varying channels. The sweep spread carrier under Doppler spreading can be expressed as [31]:

(8) $$x(t-\tau_{i})=s(t-\tau_{i})\cdot e^{j(\omega_{L}\left\{\frac{t-\tau_{i}}{T_{sw}}\right\}T_{sw}+m{\left(\left\{\frac{t-\tau_{i}}{T_{sw}}\right\}T_{sw}\right)}^{2})}\cdot e^{j\omega_{d}^{i}\left(t-\tau_{i}\right)},$$

where $\omega_{d}^{i}$ is the Doppler frequency encountered in *i*-th propagation path, which reflects the influence of Doppler effection on the received signal. The last exponent in Equation (8) can reflect time-varying phase/frequency. In this case, the $\omega_{d}^{i}$ is characterized with a time dependent function specific for *i*-th path induced. Equation (8) demonstrates that Doppler shifts belonging to different paths will not be coupled while the $\omega_{d}^{i}$ stays within certain borders; so a maximum value $\omega_{d\max}^{i}$ of the time-varying bandwidth enlargement $\omega_{d}^{i}$ does not extend a half of frequency separation space between respective multipath arrivals (e.g., $\omega_{d\max}^{i}<\frac{\omega_{d}^{i}}{2}$). In this case, every arrival stays within a definite frequency range and does not influence another frequency bands; no inter-modulation between differently varying Doppler terms belonging to different propagation paths takes place.

#### 3.1.4. Propagation Model

According to the conclusion in [32], the path loss model of underwater acoustic channel over a distance *l* with signal frequency *f* is given as:

(9) $$A(l,f)=l^{k}a{\left(f\right)}^{l},$$

where *k* is the spreading factor, a(f) is the absorption coefficient. The pass loss model shown in Equation (9) can be expressed in dB, which is given by:

(10) 10logA(l,f)=k⋅10logl+l⋅10loga(f),

where k⋅10 logl is the spreading loss; l⋅10 loga(f) means the absorption loss. The *k* is the spreading factor which describes the geometry of propagation and the values are: (1) k=2 for spherical spreading; (2) k=1 for cylindrical spreading; (3) k=1.5 for practical spreading. 

The absorption coefficient a(f) can be expressed by using Thorp’s formula, which is an empirical formula; the a(f) can be expressed as:

(11) $$10\;\log a(f)=0.11\times \frac{f^{2}}{1+f^{2}}+44\times \frac{f^{2}}{4100+f^{2}}+2.75\times 10^{-4}f^{2}+0.003,$$

Equation (11) is used for frequencies above a few hundred Hz. If the frequencies are low, then Equation (11) can be rewritten as:

(12) $$10\;\log a(f)=0.11\times \frac{f^{2}}{1+f^{2}}+0.011f^{2}+0.002,$$

Therefore, when the transmission power is *P*, the received signal power will be:

(13) $$P_{r}=\frac{P}{A(l,f)}=\frac{P}{l^{k}a{\left(f\right)}^{l}},$$

According to Equation (13), when the received signal power is equal to the receive threshold $P_{rth}$, the transmission range *r* of this AUV can be calculated based on Equation (13).

#### 3.1.5. Network Model

**Figure 8:**
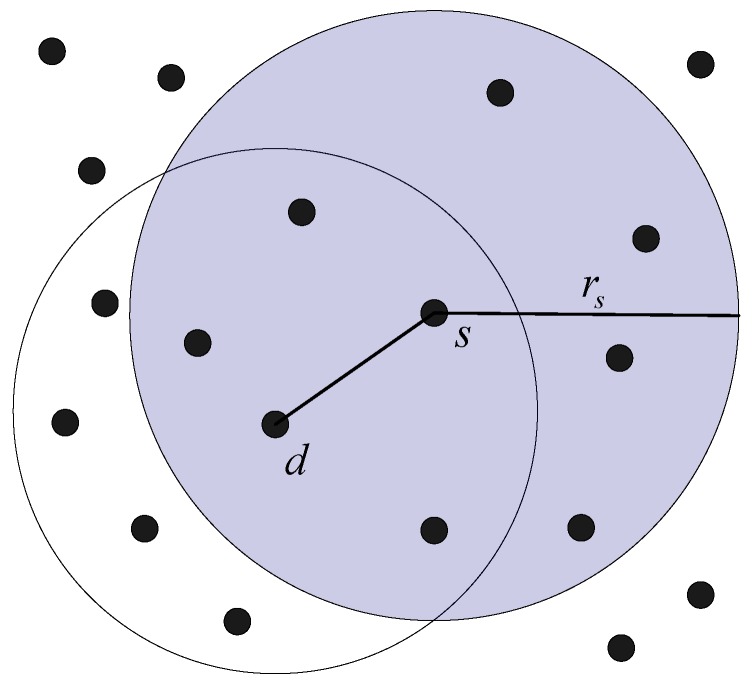
The network model of PTC algorithm.

In the use case of detection, inspection and traction of plumes, the underwater AUVs are deployed approximately in a 2-dimensional plane. An AUV can move based on a predefined path, as shown in Figure 2. Each AUV in the network can communicate with other AUVs whose distances to this AUV are smaller than its transmission range. For instance, as shown in Figure 8, AUV *s* and AUV *d* can communicate with each other when $\left\|sd\right\|\leq r_{s}$, where ‖sd‖ is the Euclidean distance between AUV *s* and AUV *d*, and $r_{s}$ is the transmission range of AUV *s*. The AUVs in network can adjust their transmission power from 0 to $P_{\max}$, which can be found in Table 2. The coverage area of AVU *s* is a circle where the centre is AUV *s* and the radius is $r_{s}$, denoted as $C(s,r_{s})$. This is shown in Figure 8. The number of one-hop neighbor AUVs in the coverage area of AUV *s* is defined as the degree of AUV *s*. For instance, in Figure 8, the degree of AUV *s* is 7.

### 3.2. Parameter Deviation Calculation

In an underwater cooperating AUVs network, the underwater AUVs are always powered by batteries. Moreover, once the energy is exhausted, the AUVs become non-functional, which has a great effect on network performance. Similarly to the energy, the buffer space of the underwater AUVs is limited, too. Thus, in case the memory space is occupied completely, the nodes cannot handle the incoming data packets, which makes the packet loss ratio increase. The occupation of the buffer space can be evaluated by queue length (in this paper, the queue length is defined as the number of data packets to be transmitted in AUV’s buffer space). Therefore, in this paper, the residual energy, the queue length, the transmission power, and the AUV’s degree will be taken into account to determine the transmission power adjustment probability for each AUV.

Based on the analysis in Section 1, we define the parameter deviation in Definition 1. The deviation of a parameter relates to the optimal solution or the constraint of this parameter.

**Definition** **1.** *The deviation of parameter x which relates to its optimal solution or constraint* $x^{*}$ *is defined as the ratio of the difference between these two values to the value of the optimal solution or the constraint, which can be expressed as:*

(14) $$D=\frac{\left|x-x^{*}\right|}{x^{*}},$$

According to Definition 1, to transmission power, when the optimal transmission power of AUV *s* is $P_{s}^{*}$ which is calculated by the optimization algorithm, the deviation of transmission power can be calculated as:

(15) $$D_{P_{s}}=\frac{\left|P_{s}-P_{s}^{*}\right|}{P_{s}^{*}},$$

Similarly to the transmission power, for the queue length (in this paper, the queue length is defined as the number of data packets to be transmitted in AUV’s buffer space), assuming that the maximum queue length allowed in AUV is $Q_{s}^{*}$, and the current queue length of AUV *s* is $Q_{s}$, then according to Equation (14), the deviation of queue length is expressed as:

(16) $$D_{Q_{s}}=\frac{\left|Q_{s}-Q_{s}^{*}\right|}{Q_{s}^{*}},$$

The total energy of AUV is $E_{s}^{*}$ and the residual energy of AUV *s* is $E_{s}$, then the deviation of residual energy is:

(17) $$D_{E_{s}}=\frac{\left|E_{s}-E_{s}^{*}\right|}{E_{s}^{*}},$$

Assuming that the needed degree of AUV for guaranteeing network connection is $n_{s}^{*}$ and the current degree of AUV is $n_{s}$, then the deviation of AUV’s degree can be calculated as:

(18) $$D_{E_{s}}=\frac{\left|n_{s}-n_{s}^{*}\right|}{n_{s}^{*}},$$

Note that in Equation (18), the AUV degree needed for guaranteeing network connections can be calculated based on the conclusion in [33]. In [33], the authors have proved that for a wireless network, if the number of neighbors of a node is larger than 5.1774 logn, then the network will be connected with probability 1; where *n* is the total number of nodes in network, so in this paper, considering the energy consumption, we choose $n_{s}^{*}=5.1774\;\log n$ as the needed AUV degree.

### 3.3. Transmission Power Adjustment Probability Calculation

When the parameter deviations have been determined, the transmission range adjustment probability can be calculated based on these deviations. The transmission range adjustment probability is defined in Definition 2.

**Definition** **2.** In an underwater cooperating AUVs network, considering the tradeoff between improving the network performance as one aspect and keeping the function of AUVs, the AUVs, in which the current transmission power does not equal the optimal transmission power that is calculated based on optimal algorithms, do not need to adjust their transmission power; rather the AUVs change their transmission power probability. This probability is called the transmission power adjustment probability.

The calculation of the transmission power adjustment probability is based on the value of the parameter deviations. The larger the deviation, the larger the probability is. Since the mathematical relationship between the transmission power adjustment probability and the parameter deviation cannot be defined clearly, in this paper, we use the fuzzy logic algorithm to calculate the transmission power adjustment probability. The input of the fuzzy logic system is the value of parameter deviation, and output is the transmission power adjustment probability of each parameters. 

As introduced in [34], the core part of fuzzy logic system is the fuzzy rules design, which decides the accuracy of the output. The more fuzzy rules are applied, the more accurate outputs are. Therefore, similarly to [34], the number of fuzzy rules used in this paper is set to 7, which are shown in Table 3.

**Figure 9:**
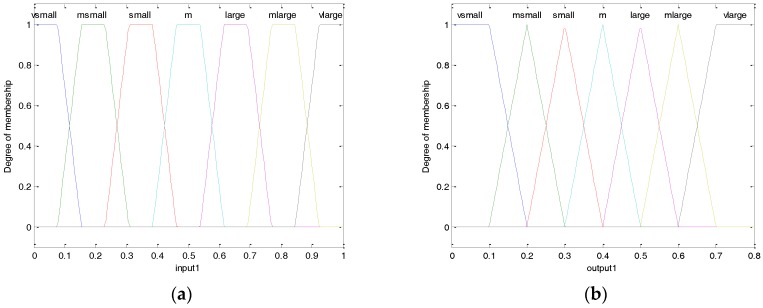
The membership functions of input (**a**) and output (**b**).

The membership functions of inputs and outputs are shown in Figure 9. 

The outputs of the fuzzy logic system are the transmission power adjustment probabilities of different parameters, which are $\rho_{P}$, $\rho_{Q}$, $\rho_{E}$, and $\rho_{n}$, respectively. However, considering the fact that for one AUV, there is only one transmission power adjustment probability, therefore, the actual transmission power adjustment probability should be determined based on these four probabilities. Moreover, for guaranteeing the network performance, the transmission power adjustment probability should be determined by the parameter in which the performance is the worst (i.e., the parameter which the deviation is the largest); this is called the Cask Principle. For instance, for $\rho_{P}$, $\rho_{Q}$, $\rho_{E}$, and $\rho_{n}$, assuming that $\rho_{Q}$ is the largest in these four probabilities, which means that the queue length is long in AUV; if the AUV chooses a probability which is smaller than $\rho_{Q}$ as the transmission power adjustment probability, then the performance of queue length cannot be guaranteed. Therefore, the approach used to decide the transmission power adjustment probability in this paper is setting the maximum probability of these four probabilities as the actual transmission power adjustment probability of AUV, which can be expressed as:

(19) $$\rho_{s}=\max\left\{\rho_{P},\rho_{Q},\rho_{E},\rho_{n}\right\},$$

Equation (19) means that the probability will be decided by the parameter which has the worst performance of all the parameters that are concerned. This approach is efficient. On the one hand, the ratio of the AUVs which need to adjust their transmission power is reduced, so the control cost of the network reduces, too; on the other hand, since the transmission power adjustment probability is decided by the parameter which has the worst performance, the network performance can be guaranteed. When the transmission power adjustment probability has been calculated, the AUVs adjust their transmission power according to this probability.

For evaluating the effectiveness of the PTC algorithm, we define the transmission power adjustment ratio for underwater cooperation AUVs network as follows.

**Definition** **3.** *The transmission power adjustment ratio is defined as the ratio of the number of AUVs which adjust their transmission power to the total number of AUVs in network, which can be expressed as:*

(20) $$R_{p}=\frac{AUVs\;adjust\;the\;transmission\;power}{total\;AUVs\;in\;the\;network}$$

According to the PTC algorithm, not all the AUVs change their transmission power when the current transmission power does not equal the optimal one. Therefore, we can conclude Theorem 1 as follows.

**Theorem** **1.** The PTC algorithm can reduce the transmission power adjustment ratio greatly.

**Proof.**  According to Equation (19), in PTC algorithm, the transmission power adjustment probability of AUV *s* is $\rho_{s}$. Assuming that there are *n* AUVs in network and the number of AUVs which the transmission power does not equal to the optimal one is $n_{s}$; therefore, the average number of AUVs which adjust their transmission power can be calculated as:

(21) $$E\left(n_{s}\right)=\sum_{i=1}^{n_{s}}\rho_{i},$$

Then according to Definition 2, the transmission power adjustment ratio of the PTC algorithm can be calculated as:

(22) $$R_{p}=\frac{E\left(n_{s}\right)}{n}=\frac{\sum_{i=1}^{n_{s}}\rho_{i}}{n},$$

However, in traditional topology control algorithms, once the transmission power does not equal to the optimal one, the AUVs need to adjust their transmission power. Since the number of AUVs which the transmission power does not equal to the optimal one is $n_{s}$, the transmission power adjustment ratio of the traditional topology control algorithm can be calculated as:

(23) $$R_{p}=\frac{n_{s}}{n},$$

Since $\rho_{i}<1$, so the $R_{p}$ in Equation (23) is larger than that in Equation (22); moreover, the smaller $\rho_{s}$, the smaller $R_{p}$ is. Therefore, the transmission power adjustment ratio in PTC algorithm is smaller than that in traditional topology control algorithm. ☐

### 3.4. PTC-Based FTC Algorithm

Based on Section 3.2 and Section 3.3, the transmission power adjustment probability of AUV can be calculated. After that, the AUVs will adjust their transmission power according to this probability.

Since many transmission power allocation algorithms have been proposed in the past decades, the transmission power allocation algorithm will be not the main research topic of this paper. In this paper, the calculation of the optimal transmission power is based on the FTC algorithm which is proposed by [6]. The FTC algorithm is the learning-based fuzzy logic control algorithm for topology control. In the following, we will introduce this algorithm briefly.

**Figure 10:**
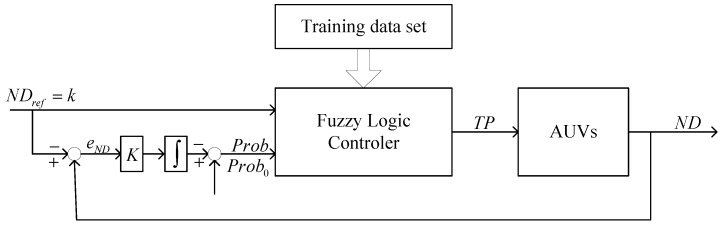
The fuzzy-logic topology control (FTC) scheme.

Figure 10 shows the system structure of FTC. Adjusting the communication power is a very common capability of many AUVs. The output of FTC is the transmission power (*TP*). The target of FTC is to reach a specific degree of AUVs. Therefore, the input is the desired AUV’s degree, denoted by $ND_{ref}$. On the other hand, according to the conclusion in [35], the probability that AUV’s degree is *n* is shown in Equation (24), so the probability that an AUV has *n* neighbors is another fuzzy logic controller input, denoted by *Prob*. In practice, $ND_{ref}$ is integer and the transmission power has an upper bound $P_{\max}$, i.e., $ND_{ref}>0$ and $0\leq P\leq P_{\max}$:

(24) $$P(ND\geq k)=f(k,P)=1-\sum_{n=0}^{k-1}\frac{{\left(\rho \pi r^{2}\right)}^{n}}{n!}e^{-\rho \pi r^{2}},$$

The training data set is provided by Equation (24); the fuzzy controller can be obtained through the neuro-adaptive learning algorithm. In Equation (24), the transmission range can be calculated based on Equation (13). The parameters of the membership function are automatically tuned through a back propagation algorithm individually or in combination with a least squares method. The generation of the training data set can be shown as follows. As illustrated in Figure 10 and Equation (24), the inputs are $ND_{ref}$ and *Prob*, and the output is the transmission power. Given ρ, $ND\in \left\{k_{1},k_{2},\ldots ,k_{m}\right\}$ and $TP\in \left\{p_{1},p_{2},\ldots ,p_{m}\right\}$, Prob=f(ND,TP) can be calculated from Equation (24). The training data set *T* is a s×3 matrix in the form of [ND,Prob,TP], where s=m⋅j. For instance, one element in the training data set is (3, 0.9, 0.25); this means that the transmission power is set to 0.25 if the probability that ND≥3 is 0.9, where the transmission power is normalized (i.e., the maximum transmission power is (1). Since *ND* is characterized by probability, it is necessary to adjust the AUV’s degree if an AUV does not reach *ND*. For instance if *TP =* 0.25 cannot actually lead to *k = ND*, then the next step is to adjust *Prob* to a higher value according to the AUV’s degree error $e_{ND}$. There is an integral controller outside the fuzzy control to adaptively change *Prob* (Figure 10). From the control theory point of view, the system properties are controlled by parameter $Prob_{0}$ and *K*. If $e_{ND}$ is less than 0, *K* is configured to be half of its initial value. Therefore, according to the FTC algorithm, the process of the PTC based FTC algorithm can be expressed as follows:

*Step* *1*: Getting the optimal transmission power $P^{*}$ based on an optimal transmission power allocation algorithm, such as the FTC algorithm [6];
*Step* *2*: Calculating the deviations of each parameter, which are $D_{P}$, $D_{Q}$, $D_{E}$, and $D_{n}$;
*Step* *3*: Calculating the transmission power adjust probabilities based on the parameter deviations calculated in Step 2 and the fuzzy logic system shown in Section 3.3; the transmission power adjustment probabilities of parameters are $\rho_{P}$, $\rho_{Q}$, $\rho_{E}$, and $\rho_{n}$;
*Step* *4*: Finding the maximum transmission power adjustment probability of $\left\{\rho_{P},\rho_{Q},\rho_{E},\rho_{n}\right\}$ and setting this probability as the transmission power adjustment probability of the AUV;
*Step* *5*: Adjusting the transmission power of the AUV based on the probability calculated in Step 4 and the optimal transmission power $P^{*}$ calculated in Step 1.

The PTC-based FTC algorithm can be found in Algorithm 1.
**Algorithm 1.** PTC-based FTC algorithm**Inputs:** Training data set, T=(k,Prob,TP);Maximum transmission power, $P_{\max}$;Reference degree of AUV, $ND_{ref}$;Initial probability, $Prob_{0}$;Initial *K*, *K_0_*;The maximum queue length, *Q*;The maximum residual energy, *E*:$TP_{i}⇐P_{\max}$;$Prob⇐Prob_{0}$;Broadcast HELLO message with current $TP_{i}$;For messages received from other AUVs, store the ID of its neighbor AUVs;Calculate the number of neighbors *ND* in the neighbor list;Calculate $e_{ND}=ND-ND_{ref}$;if $e_{ND}<0$ then  $K=K_{0}$;else  $K=\frac{K_{0}}{2}$;end if$Prob⇐Prob-K\cdot e_{ND}$;$TP_{i}⇐FTC(ND_{ref},Prob)$;Calculate the deviations $D_{P_{s}}$, $D_{Q_{s}}$, $D_{E_{s}}$, $D_{n_{s}}$;Input the deviations into the fuzzy logic system to calculate the transmission power adjustment probability $\rho_{P}$, $\rho_{Q}$, $\rho_{E}$, and $\rho_{n}$;$\rho_{s}⇐\max\left\{\rho_{P},\rho_{Q},\rho_{E},\rho_{n}\right\}$;Adjust the transmission power according to $\rho_{s}$ (random decision based on the probability value).

Note that in this paper, the PTC algorithm is combined with the FTC algorithm; however, in practice, the PTC algorithm can be combined with other different topology control algorithms to improve the performance and reduce the transmission power adjustment ratio.

## 4. Simulation and Discussion

In this section, we will evaluate the performance of PTC algorithm by simulation. To highlight the outstanding qualities of the PTC algorithm, we combine the PTC with the FTC algorithm and compare the performance of FTC algorithm with the PTC-FTC algorithm. The simulation results shown in [6] have demonstrated that the FTC algorithm is highly efficient in controlling the network topology.

### 4.1. Simulation Configuration

Based on the communication architecture of SWARMs project shown in Section 3.1, the simulation configuration parameters of the underwater cooperating AUVs network is shown in Table 4.

### 4.2. Simulation Results

**Figure 11:**
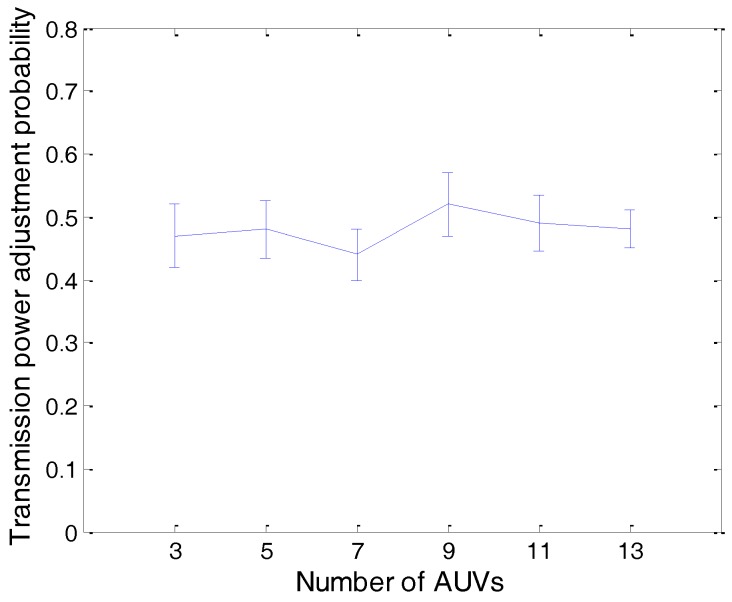
The average transmission power adjustment probability of PTC-FTC.

**Figure 12:**
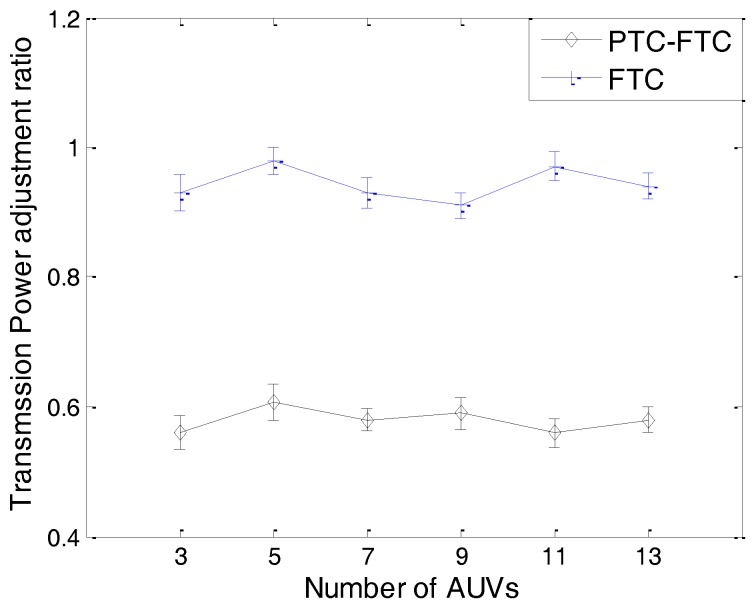
The average transmission power adjustment ratio of PTC-FTC and FTC.

**Figure 13:**
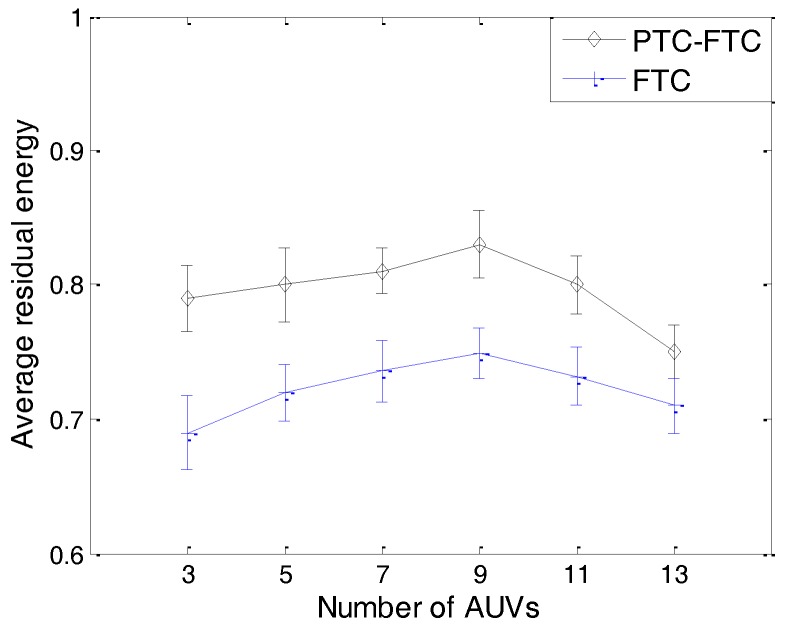
The average residual energy of PTC-FTC and FTC.

**Figure 14:**
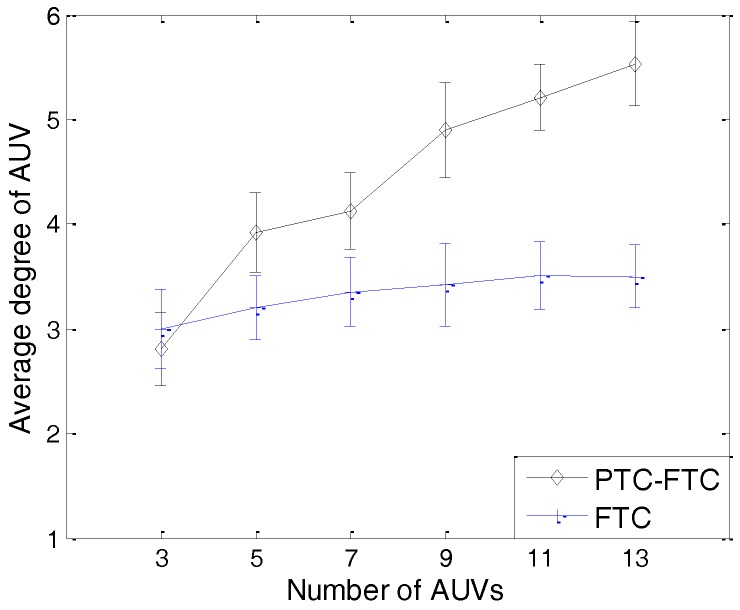
The average node degree of PTC-FTC and FTC.

**Figure 15:**
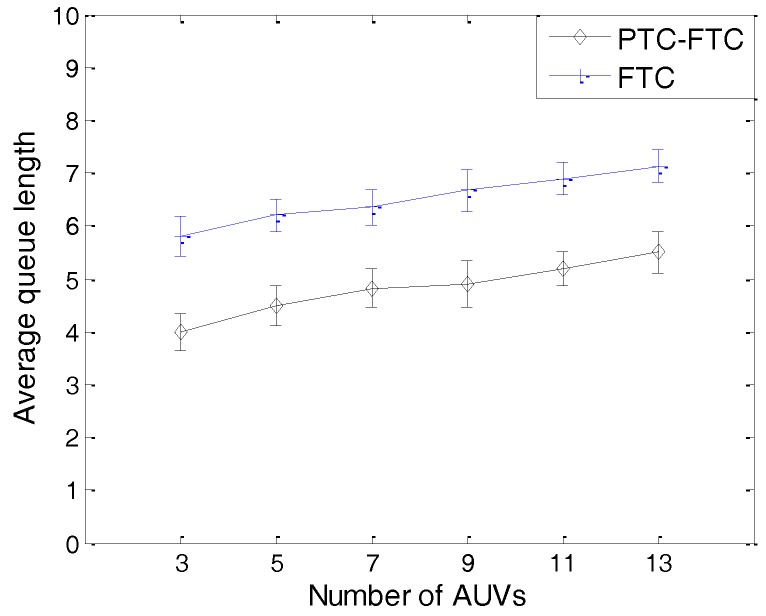
The average queue length of PTC-FTC and FTC.

The simulation results can be found from Figure 11, Figure 12, Figure 13, Figure 14 and Figure 15. The simulation tool is DESER, which is an extension toolbox based on the NS-2 simulator. The simulation parameters can be found in Section 3.1 and Section 4.1.

In Figure 11, the average transmission power adjustment probability of the AUV is shown. From this figure we can find that this probability varies between 0.4 and 0.5. When the number of AUVs increases, there is no evidence showing that the transmission power adjustment probability increases too. This is because the probability is determined by the residual energy, the transmission power, the node degree, and the queue length jointly, so an increasing of the number of AUVs in the network cannot affect the probability greatly.

For instance, when the number of AUVs increases, the transmission power decreases and the residual energy increases, which can be found in Figure 13; however, due to the increase of the AUV’s degree, the queue length will increase (shown in Figure 15); therefore, the probability may not increase. The transmission power adjustment ratio can be found in Figure 12. 

We can see in Figure 12 that the transmission power adjustment ratio of the PTC-FTC algorithm is much smaller than that of the FTC algorithm. The transmission power adjustment ratio in the FTC algorithm is about twice larger than that in the PTC-FTC algorithm. This demonstrates that the PTC algorithm is efficient at reducing the transmission power adjustment ratio. Similarly to the transmission power adjustment probability shown in Figure 11, the transmission power adjustment ratio does not increase with the increasing number of AUVs in the network. The reason is that when the number of AUVs in the network increases, the number of AUVs which need to adjust their transmission power increases too; moreover, according to the dynamics of an underwater cooperating AUVs network, there is no evidence that shows that the increase of the number of AUVs which need to adjust their transmission power is proportional to the increase of the total number of AUVs in the network. The notable properties of PTC-FTC algorithm also can be seen in Figure 13, Figure 14 and Figure 15. In Figure 13, the residual energy of an AUV is presented. The residual energy in the PTC-FTC algorithm is larger than that in the FTC algorithm. With an increasing number of AUVs in the network, the residual energy increases when the number of AUVs in the network is smaller than nine and decreases when this number is larger than nine, both in the PTC-FTC algorithm and the FTC algorithm. This conclusion is easily understood. When the number of AUVs is smaller, in order to guarantee the network connections, the transmission power of each AUV in network is larger and the network interference, the retransmission, and the network competition are smaller; moreover, when the number of AUVs in the network is small, the transmission power will play a dominant role in the energy consumption performance, and when this number increases, the transmission power will decrease, so the residual energy increases when the number of AUVs is smaller than nine. However, when the number of AUVs is larger, the dominant parameters will be the network interference, the retransmission, and the network competition, so when the number of AUVs in network increases, the residual energy decreases. 

As mentioned in Section 3.2, the needed degree of AUVs in the PTC-FTC algorithm is dynamic and defined based on the conclusion in [33]; moreover, to get a fair simulation result, the AUVs’ degree in the FTC algorithm is set to three, which is the same as that shown in [6]. The simulation results in Figure 14 illustrate this simulation setting. In Figure 14, when the number of AUVs in the network increases, the AUVs’ degree of the PTC-FTC algorithm increases fast to guarantee network connectivity; moreover, the AUVs’ degree of the FTC algorithm remains stable. When the number of AUVs in the network is less than three, the AUVs’ degree in the PTC-FTC algorithm is smaller than that in the FTC algorithm. This can be explained by the different needed degree calculation algorithms used in the PTC and FTC. In the FTC algorithm, this needed degree is fixed; however, in the PTC algorithm, according to the conclusions in [33], this number is dynamic according to the different network conditions. Interesting conclusions can be found when comparing the simulation results in Figure 13 with those in Figure 14. In Figure 14, the average degree of an AUV in the PTC-FTC algorithm is larger than that in the FTC algorithm; however, the residual energy of the PTC-FTC algorithm is larger than that of the FTC algorithm. This can be explained by the conclusion in Figure 12. Since the probability adjustment ratio in the PTC-FTC algorithm is smaller than that in the FTC algorithm, the energy consumption for topology control and retransmission caused by network competition is smaller than in the FTC algorithm, so even though the transmission range in the PTC-FTC algorithm is larger than that in the FTC algorithm, the residual energy in the PTC-FTC algorithm is large. 

As shown in Figure 15, the queue length of the PTC-FTC algorithm is much smaller than that of the FTC algorithm, and with an increasing number of AUVs in the network, the queue lengths in both the PTC-FTC algorithm and FTC algorithm increase. This is because the more AUVs in network, the more data packets need to be transmitted; therefore, the queue length will increase when the number of AUVs increases. Additionally, due to the fact the transmission power ratio of the PTC-FTC algorithm is much smaller than that of the FTC algorithm (which can be seen in Figure 11), the control messages in the PTC-FTC algorithm are much smaller than in the FTC algorithm, which means the queue length in PTC-FTC algorithm is small, too. 

The results presented from Figure 11, Figure 12, Figure 13, Figure 14 and Figure 15 have demonstrated that the PTC-FTC algorithm is efficient on improving network performance while reducing energy consumption for underwater cooperating AUV networks.

## 5. Conclusions

In this paper, we propose a probabilistic topology control (PTC) algorithm for underwater cooperating AUV networks which are associated with limited communication capability and high mobility. In PTC, to reduce the transmission power adjustment ratio of the topology control algorithm, when the AUVs’ transmission power does not equal the optimal transmission power calculated based on an optimal algorithm, the AUVs must not adjust their transmission power. The AUV calculates the deviation of the transmission power, the residual energy, the degree of AUV, and the queue length firstly; then the AUV calculates the transmission power adjustment probabilities of each parameter based on these deviations. The maximum probability will be chosen as the transmission power adjustment probability of the AUV. Through this approach, the transmission power adjustment ratio of the topology control algorithm can be reduced greatly (by about 40%). Since the transmission power adjustment ratio has been reduced, the network performance also improved remarkably. 

We note that in this paper, the PTC algorithm is combined with the FTC algorithm; however the PTC algorithm could also be combined with topology control algorithms other than the FTC algorithm to reduce the transmission power adjustment ratio. Additionally, while in this paper, the parameter selection is based on the requirements of the SWARMs project, these parameters are not fixed, and they can be easily changed based on the requirements of different applications. This flexibility represents one of the main advantages of PTC algorithm.

## Figures and Tables

**Table 1:** Parameters of the AUVs used in the SWARMs project.

AUV	Length (m)	Width (m) (Circular)	Height (m) (Circular)	Weight (kg)
ALISTER 9	2	0.22	0.22	70
IXN	1.9	0.5	0.3	150
Naiad	0.84	0.6	0.25	30

**Table 2:** Parameters of the S2CR communication module.

Items	Parameters
Operating depth	200 m to 2000 m depending on the housing material; 6000 m for Ti
Operating range	3500 m
Frequency band	18–34 kHz
Transducer beam pattern	Horizontally omnidirectional
Acoustic connection	(1) Burst data mode: up to 13.9 kbit/s in good channel conditions; 2.2–3.2 kbit/s in complex channel condition shallow water; (2) instant message mode: 1 kbit/s
Bit error rate	Less than 10^−10^
Power	Stand-by mode: 2.5 mW; listen mode: 5–285 mW; receive mode: ≤1.3 W; transmission mode: 2.8 W (1000 m), 8 W (2000 m), 35 W (3500 m), 80 W (max available)
Power supply	External 24 VDC; internal rechargeable battery
Dimensions	diameter 110 mm; total length 265 mm

**Table 3:** The Fuzzy rules.

INPUT (If): Parameter Deviation	OUTPUT (Then): Adjustment Probability
Very small	Very small
Medium small	Medium small
Small	Small
Medium	Medium
Large	Large
Medium large	Medium large
Very large	Very large

**Table 4:** Simulation configuration.

Parameters	Value
Simulation tool	DESERT ^1^
Simulation area	3000 m × 3000 m
Number of AUVs	3, 5, 7, 9, 11, 13
Depth	20 m
Water temperature (July)	12 °C
Water salinity (July)	18 g/L
Sound speed (July)	1475 m/s
Initial transmission range	1000 m
Transducer beam pattern	Horizontally Omni-directional
Data rate	3 kbit/s
Bit error ratio	10^−10^
Receive power	1.3 W
Transmission power	2.8 W
Channel model	Multi-path + Doppler spreading
Carrier	Sweep spread carrier (S2C)

^1^ DESERT: http://nautilus.dei.unipd.it/desert-underwater.
